# The Museum as a Mindful Space: Reducing Visitors’ Stress and Anxiety Levels Through the ASBA Protocol

**DOI:** 10.3390/bs16010116

**Published:** 2026-01-14

**Authors:** Annalisa Banzi, Pier Luigi Sacco, Maria Elide Vanutelli, Claudio Lucchiari

**Affiliations:** 1 Department of Philosophy “Piero Martinetti”, Università degli Studi di Milano, 20122 Milan, Italy; claudio.lucchiari@unimi.it; 2 Department of Philosophical, Pedagogical and Economic-Quantitative Sciences, Università Degli Studi Chieti-Pescara “Gabriele d’Annunzio”, 65127 Pescara, Italy; pierluigi.sacco@unich.it; 3 Department of Psychology, University of Milano-Bicocca, 20126 Milan, Italy; maria.vanutelli@unimib.it

**Keywords:** ASBA, mindfulness, museum, wellbeing, stress, anxiety, mental health intervention, MBSR

## Abstract

Active involvement in creative activities, known as creative health, has been shown to enhance wellbeing, with museums serving as unique spaces for health promotion; however, visitors often require guidance to derive significant benefits from these institutions. This study, part of the larger ASBA (Anxiety, Stress, Brain-friendly museum Approach) project, evaluates the first phase of an intervention specifically focused on a Mindfulness protocol adapted to museum contexts. It has employed a single-group pre–post design with 79 healthy adults recruited from the non-clinical population. Participants were involved in a 15 min standardized mindfulness practice adapted from Mindfulness-Based Stress Reduction (MBSR) in either an art or science museum. State anxiety (SAI) and mood (VAS) were assessed at baseline and post-intervention, alongside personality traits (BFI-10) and interest measures to identify individual moderators of treatment response. The practice appeared to reduce state anxiety significantly in both settings, with large effect sizes. Specific moderators emerged: openness to experience predicted anxiety reduction in the art museum, whereas science interest predicted outcomes in the science setting. These findings suggest that brief, standardized mindfulness protocols implemented through the ASBA framework can provide promising immediate benefits for visitor wellbeing across diverse museum environments.

## 1. Introduction

According to the American Psychological Association (2023), stress is the physiological and psychological response to internal or external stressors. It involves changes that affect almost every system in the body, influencing the way people feel and behave. By causing these mind–body changes, stress directly contributes to psychological and physiological disorders and illness, reducing the quality of life (Agorastos & Chrousos, 2022).

Anxiety, on the other hand, is an emotion characterized by apprehension and somatic symptoms of tension whereby an individual anticipates imminent danger, catastrophe, or misfortune (Szuhany & Simon, 2022). Chronic anxiety may have severe effects on physical and psychological wellbeing, increasing the perception of stress and decreasing quality of life in the long term (Kudryavtseva, 2021).

Levels of psychosocial distress in society are significant, as evidenced by the widespread use of prescribed antidepressants and days of work lost due to stress and anxiety (Hassard et al., 2021). The COVID-19 epidemic has further exacerbated these conditions (Mahmud et al., 2021). However, active involvement in creative and cultural activities offers a wide range of benefits, such as promoting wellbeing, quality of life, and health.

Various longitudinal studies on very large samples have shown a positive association between being involved in cultural activities (such as attending museums regularly) and various physical and mental health and wellbeing outcomes, including life expectancy (e.g., Bygren et al., 1996; Cuypers et al., 2012; Fancourt & Steptoe, 2019; Rapacciuolo et al., 2016; Węziak-Białowolska & Białowolski, 2016).

Addressing these challenges requires expanding public health strategies beyond traditional biomedical interventions toward more holistic, preventive approaches. Creative health, defined by the World Health Organization (World Health Organization [WHO], 2019) and the UK’s All-Party Parliamentary Group on Arts, Health and Wellbeing (2017) as the integration of creative and cultural activities into health promotion and disease prevention, represents an instance of such a paradigm shift. This framework encompasses visual and performing arts, crafts, music, literature, and nature-based creative activities, positioning cultural participation not as a form of leisure that remains peripheral to health, but as a fundamental determinant of wellbeing alongside nutrition, physical activity, and social connection (Fancourt & Finn, 2019).

The theoretical foundation of creative health draws heavily on salutogenic models of health, which emphasize building resources that promote movement toward health rather than solely treating pathology (Antonovsky, 1996). This perspective aligns with contemporary public health recognition that approximately 80% of health determinants lie outside clinical settings (Sonke et al., 2019), residing instead in the social, environmental, and cultural conditions of daily life. Creative health interventions address these determinants by fostering a sense of coherence, social connection, emotional expression, cognitive stimulation, and meaning-making, that is, psychological resources that buffer against stress and support resilience.

Operationally, creative health integration into formal health systems occurs through social prescribing mechanisms, whereby healthcare providers refer patients to community-based cultural and creative activities as complements to or alternatives for clinical treatment (World Health Organization [WHO], 2022). First developed in the UK and now implemented across Europe, Canada, and Asia, social prescribing recognizes that many health needs, particularly those related to chronic stress, social isolation, and mild-to-moderate mental health symptoms, may be more effectively addressed through community engagement than pharmaceutical or psychotherapeutic interventions alone (H. J. Chatterjee et al., 2018). Systematic reviews confirm that arts-based interventions produce measurable improvements in anxiety, depression, social isolation, and quality of life (Fancourt & Finn, 2019), establishing creative health as an evidence-based public health strategy rather than a merely aspirational policy.

Museums have increasingly been recognized as privileged locations where people can care for themselves (H. Chatterjee & Noble, 2016). Recent systematic reviews have shown that museum-based interventions can significantly impact mental health recovery (Goodman-Casanova et al., 2023). Some studies have explored the exhibits’ capacity to produce positive experiences, often in laboratory settings (e.g., Kemp & Cupchik, 2007; Vessel et al., 2012). Other studies have focused on the museum environment itself, highlighting it as a place with distinctive emotional, cognitive, and social characteristics that can promote relaxation, wellbeing, and social life (e.g., Tinio et al., 2015; Grossi et al., 2019).

Museums are now committed to promoting wellbeing in their communities in various ways (Brown, 2019) and many of them are also willing to experiment with additional strategies to achieve this goal (Šveb Dragija & Jelinčić, 2022). As of 2018, Canadian family physicians can prescribe museum visits to improve physical and mental wellbeing. In 2021, Belgium launched a three-month pilot experiment based on the Canadian example. Brussels doctors began prescribing museum visits to treat COVID-19-related stress and anxiety, involving five museums and one hospital (Huckaby, 2021). These initiatives reflect a growing recognition of museums’ potential role in healthcare, as documented in recent reports by the American Alliance of Museums (2024) and the World Health Organization acknowledgment of arts and culture in mental health recovery (Goodman-Casanova et al., 2023).

The main difficulty museums can face is providing activities to relieve stress and anxiety through encounters with exhibits during visits. It cannot be assumed that museumgoers can connect with and benefit from museum spaces and objects without proper guidance. At present, there are no specific protocols directed at treating stress and anxiety in museums. There is an urgent need for evidence-based, standardized interventions that can be implemented and tested across different museum contexts to make results comparable.

The ASBA (Anxiety, Stress, Brain-friendly museum Approach) project is based on a research protocol approved by the Ethics Committee of the University of Milano-Bicocca (protocol code: 733/22). The project’s dual objective is to enhance visitors’ wellbeing while promoting the value of museum collections and mission (Banzi, 2022; Lucchiari et al., 2024). Specifically focused on addressing stress and anxiety, the ASBA framework is designed for easy adaptation across diverse museum environments. The project targets a non-clinical population seeking cultural experiences that improve wellbeing by reducing stress and anxiety levels; it encompasses several methods to be tested, including Mindfulness, Art Therapy, Visual Thinking Strategies, and Nature + Art.

In this paper, we report the results of the first phase of the project, which focused on a Mindfulness protocol specifically adapted to the museum context, establishing a deep connection between visitors and the objects displayed.

Mindfulness-Based Stress Reduction (MBSR) interventions, developed by Kabat-Zinn (1990), possess a robust evidence base demonstrating their efficacy across diverse populations and settings, with consistent moderate to large effect sizes for anxiety and stress reduction in both clinical and non-clinical groups (Khoury et al., 2015; de Vibe et al., 2017; Galante et al., 2023). Recent findings further confirm that these interventions are particularly effective for anxiety (Sparacio et al., 2024), performing as well as pharmacological treatments (Hoge et al., 2023). In the cultural sector, Mindfulness allows visitors to explore museums and collections through a new lens, promoting reflection and observation. Research has shown that this practice is effective within museums, stimulating an appropriate approach to cultural heritage that aligns with the museum mission (e.g., Echarri & Urpi, 2018). Recent studies specifically highlight how Mindfulness-based approaches in museums can significantly reduce participants’ anxiety and stress and cultivate deeper connections with the artworks (Filipowska et al., 2024; Chan et al., 2024).

In particular, the goals are to create an MBSR-adapted protocol suitable for museum implementation; assess the feasibility and acceptability of this protocol across diverse museum settings; evaluate preliminary efficacy through changes in state anxiety and wellbeing measures; and analyze individual difference factors, such as personality traits and cultural interests, that may help predict treatment response.

Based on the established efficacy of MBSR interventions, the potential of museums as health-promoting environments, and theoretical considerations about person–environment fit, we tested the following hypotheses:

**H1** (Primary hypothesis)**.** *The MBSR-adapted mindfulness protocol will significantly reduce state anxiety from pre-intervention to post-intervention in both museum settings, as measured by the State Anxiety Inventory (SAI).*

**H2** (Secondary outcomes)**.** *The intervention will significantly improve wellbeing indicators, including mood, stress, contentment, calmness, and restlessness, as measured by Visual Analogue Scales (VAS).*

**H3** (Context-specific predictors)**.** *Individual characteristics will predict intervention response differently across museum types, specifically:*

**H3a.**  *In the art museum, openness to experience will predict greater anxiety reduction, as this personality trait is associated with esthetic receptivity, cognitive flexibility, and capacity for absorption, all characteristics that have been theorized to facilitate engagement with complex visual artworks during mindful observation (Fayn et al., 2015; Barbieri et al., 2024; Palumbo et al., 2025).*

**H3b.**  *In the science museum, interest in science will predict greater anxiety reduction, as domain-specific knowledge and intellectual curiosity are theorized to facilitate deeper engagement with scientific exhibits, providing meaningful cognitive scaffolding for sustained mindful attention (Powell et al., 2016; Wu & Wu, 2020; Sekiguchi, 2023; Taheri et al., 2024).*

**H4** (Differential effects)**.** *Museums will produce differential effects on specific wellbeing dimensions based on their distinct affordances. Specifically,*

**H4a.**  *Art museums, featuring emotionally evocative figurative art, will produce stronger affective engagement.*

**H4b.**  *Science museums, featuring analytically oriented natural history content, will produce greater improvements in mental clarity, reflecting more focused, cognitively stable mindsets.*

This protocol development study prioritizes feasibility demonstration, standardization, and preliminary efficacy evaluation, providing the foundation for future randomized controlled trials examining causal mechanisms, long-term outcomes, and comparative effectiveness against alternative interventions.

## 2. Methods

### 2.1. Design

This study employed a single-group pre–post design to develop and conduct preliminary testing of a standardized Mindfulness protocol for museum settings. The research represents an intervention development study consistent with Stage 1b of the NIH Stage Model for behavioral intervention research (Onken et al., 2014), focusing on three primary objectives: protocol development and standardization; feasibility assessment across different museum types; and preliminary efficacy evaluation through validated outcome measures.

The Mindfulness protocol (detailed in Section 2.3.3) was developed collaboratively by a certified MBSR instructor, museum staff, and researchers and then refined through pilot testing. This Mindfulness protocol development effort addresses a critical gap in the museum-health literature, where interventions have frequently lacked standardization, detailed documentation, or replicability across different settings. All sessions were delivered by the same certified instructor using a standardized script to ensure consistency.

The study was conducted in two museum contexts: the Modern Art Gallery (MAG; Milan, Italy), featuring Italian Romantic paintings and sculptures, and the Natural History Museum (NHM; Milan, Italy), featuring tropical nature dioramas with diverse flora and fauna. This dual-museum strategy serves complementary research objectives. First, cross-context implementation allows us to assess protocol robustness: if the same standardized procedure yields comparable anxiety reduction effects in fundamentally different museum environments (art vs. science), this provides preliminary evidence of generalizability and suggests that the protocol is able to capture core therapeutic mechanisms transcending specific content domains. Second, a separate analysis of each setting enables the identification of context-specific factors that predict intervention response. Art and science museums attract different audiences, activate different cognitive and emotional processes, and afford different types of engagement with cultural material. Rather than asking “which museum is more effective?”, a question with limited practical value given that museums serve different populations and purposes, we ask “what visitor characteristics predict better outcomes in each context?”

The study was designed to generate preliminary efficacy data sufficient to justify investment in future randomized controlled trials; provide effect size estimates for power calculations in subsequent studies; identify relevant moderator variables for stratification or subgroup analyses; and offer museums an evidence-informed, replicable protocol ready for broader implementation and evaluation (Lucchiari et al., 2024). Our approach provides actionable guidance for museums implementing the protocol.

### 2.2. Participants

79 healthy adults with a mean age of 46.49 years (SD = 16.7), ranging from 18 to 73 years, participated in the experiment (see Table 1) at either the MAG NHM. Of these, 44 participants visited the MAG, and 35 participants visited the NHM. Regarding museum habits, more than 54% of participants reported visiting museums more than 6 times a year, while 21.5% and 19% reported attendance of 3–5 and 1–2 times per year, respectively. Only 5.1% reported never visiting a museum throughout the year.

A convenient sample was recruited within the general citizenry rather than through specific museum visitors or university courses, ensuring the inclusion of individuals from diverse ages and various social, economic, and cultural backgrounds. This was achieved through a dedicated recruitment procedure and the collaboration of numerous entities, with the specific goal of reaching people with varying attitudes and habits regarding museum attendance. Furthermore, the entire procedure was standardized to guarantee repeatability, although controlling for all involved psycho-social variables remains a challenge. The call for participants was distributed via social media, newsletters, and the websites of the participating partners. We spread information about the project on very different physical and online sites in order to reach a heterogeneous population. Thus, we have limited the sharing of information through canonical university or museums recruitment strategies, and we relied on the support of many alternative channels, such as magazines, newspapers, social media, email lists and word of mouth. Our study has been presented in various institutional and educational events, allowing us to network to various engagement opportunities. Consequently, our sample includes few university students and habitual visitors of the museums taking part in the study, allowing us to gather data on people with different age, experiences, values, and preferences.

Each month, prior to the start of the sessions, the registration calendar was made available to the public through a dedicated website as new slots became available. Registration closes the day before each session to allow researchers to verify participant eligibility and ensure the completeness of the data. Through the dedicated web page of the project, participants scheduled their visit dates and accessed the informed consent form. This document explained all the procedures in detail, along with the objectives and the risks of the study. It also included a detailed section clearly stating the participant’s right to withdraw from the study at any stage, along with the privacy policy. After agreeing to these terms, participants completed the screening questionnaire to gather personal data and verify inclusion and exclusion criteria. Participants then filled out the psychological traits questionnaires and were assigned to a specific study (see the Procedure Section 2.3).

#### 2.2.1. Inclusion Criteria

- Being 18 years of age or older.
- Accepting the conditions for participation and having signed the consent form.

#### 2.2.2. Exclusion Criteria

- Inadequate language skills to understand verbal instructions and interacting with presenters and peers.
- Current diagnosis of a disease of psychiatric or neurological interest.
- Uncorrected visual or hearing impairment.
- Any other medical condition that could negatively affect the activities to be undertaken.
- Prior experience in Mindfulness (>3 months of regular practice).

Exclusion criteria reflect the study’s targeting of the general population and not of people with clinical conditions. Since the procedures involved group activities, we could not provide individualized support and then we had to exclude people with language or sensorial impairments that could prevent full participation in the proposed activities. Furthermore, in agreement with our mindfulness expert, we chose to exclude people with a sound experience in mindfulness, since this could limit the generalizability of results. Three months is considered by experts as a minimum period to develop an expertise in mindfulness practice.

Inclusion and exclusion criteria were assessed initially using an online self-report questionnaire, and the procedure was stopped whenever any reported information implied unfeasibility to participate. Consequently, only responders who passed all the criteria were admitted to the study. A second check was then implemented before the museum experience. Only participants who successfully went through the screening questionnaire and reserved a seat for a given date were admitted at a museum session. Here, a researcher had a brief individual interview with each participant in order to double-check the information provided online and to confirm that the informed consent was understood and accepted.

### 2.3. Procedure

**Figure 1:**
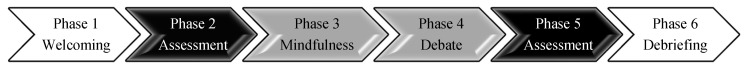
Graphic representation of the experimental procedure. The Figure shows the six phases: (1) Welcoming and consent verification, (2) Pre-treatment assessment (SAI, VAS), (3) 15 min Mindfulness practice in museum room, (4) Group discussion with museum staff, (5) Post-treatment assessment, and (6) Debriefing and information sharing.

We describe the whole study procedure, which involved 6 consecutive phases (see Figure 1).

#### 2.3.1. Procedure Phase 1: Welcoming

On the day of the session, participants were welcomed in a designated area of the museum, where a check-in was conducted to verify that the questionnaires were completed and the consent forms signed. Once all participants for the session had arrived, they were led to a room reserved for the study presentation. In this setting, the researchers reiterated key study information, introduced the instructors and the other researchers involved, and provided a brief overview of the Mindfulness technique.

#### 2.3.2. Procedure Phase 2: Pre-Treatment Assessment

Pre-treatment questionnaires were administered, which were designed to measure state anxiety levels (SAI) and mood (VAS), together with other baseline measurements (see below). Participants could choose any seat in the large hall to encourage comfort and self-confidence.

#### 2.3.3. Procedure Phase 3: Mindfulness Practice

Participants were led to a museum room specifically selected for the Mindfulness activity. Chairs were pre-arranged in the room to ensure each participant could focus on significant elements of the objects displayed. Participants were invited to take a seat, after which the Mindfulness practice was conducted, lasting approximately 15 min (see below for a full description of the protocol).

#### 2.3.4. Procedure Phase 4: Inquiry

Following the practice, the chairs were arranged in a circle to facilitate group discussion. During this phase, participants freely shared their feelings and opinions regarding their experience, including which museum objects (or specific details) they had focused on. The Mindfulness instructor and a member of the museum staff facilitated the conversation, providing feedback on the practice and the characteristics of the exhibits. This allowed participants to deepen their understanding and acquire new knowledge about the objects on display.

#### 2.3.5. Procedure Phase 5: Post-Treatment Assessment

At the conclusion of the discussion, participants were escorted back to the conference room to complete the post-treatment questionnaires. These were identical to the pre-experience versions, with the addition of a final section where participants could provide general comments on their experience.

#### 2.3.6. Procedure Phase 6: Debriefing

At the end of the post-experience questionnaires, participants could ask further questions to the instructors and researchers, as well as receive further information on future developments of the study, or where to find information for further investigation.

Before the beginning of the experimental sessions, a pilot session was performed including all these phases to check that all the stages were properly constructed.

### 2.4. Materials

The study involved the use of screening, trait, and state measures. A screening questionnaire was administered to collect individual information (age, gender, experience and preferences with museums and with the techniques used in the project). Trait measures are intended to assess the participant’s baseline characteristics and include trait anxiety, perceived stress in the last month, and personality traits.

State measures, on the other hand, are designed to assess the impact of treatment, as their value is expected to vary over a short period of time. The main variable in the study is state anxiety, which is measured before and after the museum session. Similarly, other psychological dimensions are measured using Visual Analog Scales.

#### 2.4.1. Screening Questionnaire

The questionnaire included questions about inclusion and exclusion criteria, gender (female, male, other, prefer not to say), age, interest in art or science museums (on a Likert scale from 1: not at all, to 10: very much), and annual attendance at any type of museum (4 choice ranges: never; 1–2 times; 3–5 times; >6 times).

#### 2.4.2. Trait Measures

The State and Trait Anxiety Inventory (STAI; Spielberger et al., 1970) is the most widely used tool in the scientific literature for the psychometric measurement of anxiety. The theory of state and trait anxiety, which distinguishes between current anxiety and readiness for anxious reaction as a personality trait, is now well-supported by both clinical evidence and numerous experimental studies. The brevity of the questionnaire and the formulation of the items in terms that can be easily and immediately understood by anyone makes it very simple to administer and ensures good reliability of the scores obtained. The STAI questionnaire consists of two separate scales to measure two distinct anxiety constructs: anxiety as State (SAI) and anxiety as Trait (TAI). Both scales consist of 20 statements where participants are asked to describe how they generally feel on a Likert scale ranging from 1 (not at all) to 4 (very much). In the recruitment questionnaire, just the TAI was administered. The SAI, instead, was applied during the pre/post-assessment phases (see Section 2.4.3).

The Perceived Stress Scale (PSS; Cohen et al., 1983) is the most widely used psychological instrument for measuring the perception of stress. It is a measure of the degree to which situations in a person’s life are appraised as stressful. The items were constructed to assess the degree to which people find their lives unpredictable, uncontrollable, or overloaded. The scale also contains a series of direct questions about current levels of perceived stress. Both the items and the response options are easy to understand. In addition, the questions are general and thus free of content specific to any subpopulation. The PSS questions cover feelings and thoughts related to the last month. For each item, people are asked to indicate how often they felt a certain way, from 0 (never) to 4 (very often). Scores higher than 27 are considered high, while people with a score lower than 14 are considered to have low stress.

We also used a subjective evaluation of one’s stress level over the last month, measured on a scale from 0 to 10, in order to obtain a more comprehensive assessment of perceived stress.

BFI-10: the Italian short version (Guido et al., 2015) of the Big Five questionnaire (BFI-10) is a 10-item version (Rammstedt & John, 2007) of the more comprehensive BFI questionnaire (John et al., 1991) that contains 44 items. Participants are required to indicate their level of agreement on a scale ranging from 1 (“I do not agree at all”) to 5 (“I absolutely agree”). This questionnaire was developed to provide information about personality in an extremely short time. Indeed, the BFI-10 allows the 5 personality traits to be assessed through only two items per dimension. Previous research has clearly shown that the BFI-10 possesses psychometric properties that are comparable in size and structure to those of the BFI in its standard version (Rammstedt et al., 2020). The BFI-10 assesses 5 traits:

Openness: a person’s degree of creativity, curiosity, depth of thought, and taste for diversity. Those with low scores favor regularity, tradition, and pragmatism; those with high scores value novelty, abstract thought, and art.

Agreeableness: a person’s disposition toward kindness, cooperation, compassion, and reliability in social situations. Low agreeableness is linked to being more competitive, doubtful, and self-interested; high agreeableness is linked to being sympathetic and helpful.

Conscientiousness: a person’s propensity for self-control, planning, caution, and goal-oriented behavior. A high score indicates someone who is dependable and hardworking, has interest in interacting with the outside world, and is chatty, gregarious, active, and forceful. People that score low (introverts) favor solitude and introspection; highly extraverted individuals are stimulated by groups and social interaction.

Neuroticism: a person’s propensity to feel unpleasant feelings like stress, anxiety, worry, and emotional instability. A low score denotes a serene, safe, and resilient temperament; a high score denotes excessive emotional reactivity and mood swings.

#### 2.4.3. State Measures

SAI: to assess the changes in State Anxiety, the SAI scale was administered both before and after the Mindfulness practice (pre- and post-experience assessment) following a pre/post-protocol similar to Binnie (2010), who reported positive results after a museum visit. The scale can detect short-term changes in anxiety levels thanks to its good psychometric properties, as shown in many previous studies (e.g., Field et al., 2013; Mackay & Neill, 2010; Vitasari et al., 2011). Recent studies have employed SAI in detecting changes following Mindfulness interventions (Tang et al., 2023; Sun et al., 2025). As mentioned above, the scale includes 20 statements where participants are asked to describe how they feel in the present moment on a Likert scale ranging from 1 (not at all) to 4 (very much).

MOOD-VAS: six visual-analog scales (VAS) were presented to assess participants’ moods and states of mind before and after Mindfulness sessions. The first question was about general mood, i.e., “How do you rate your mood right now?” with a choice among 10 steps from 1 (totally negative) to 10 (totally positive). The next 5 questions required assessing the intensity of certain states of mind experienced at the present moment, from 1 (absent state) to 10 (very present state), specifically stress, mental clarity, contentment, calmness, and restlessness. VASs have been used as measures of mood change following Mindfulness interventions (Keng et al., 2013, 2017).

#### 2.4.4. Mindfulness Protocol

The Mindfulness sessions were led by a certified Mindfulness-Based Stress Reduction (MBSR) instructor. This certification ensures adherence to international standards for MBSR instruction, requiring completion of professional training and supervised teaching experience. Participants, seated in chairs, were guided in a 15 min focused-attention practice, in which participants were invited to bring their full attention to a specific stimulus. In this case, stimuli were both internal and external. Specifically, in the first phase, participants were invited to find the correct position for the practice, with their feet firmly on the floor, their backs erect and spaced from the back of the chair, and their eyes closed, to facilitate the perception of bodily sensations and breathing. The general instruction for the practice was to remain engaged in the present moment, and to gently and nonjudgmentally welcome and let go of all distracting thoughts. The instructor reminded the participants of this important aspect several times throughout the practice. In a second phase, participants were asked to attend to the body sensations associated with the contact points, feet on the floor, and buttocks on the seat, and to pay attention to their breath. In the third phase, participants were asked to open their eyes and observe a detail of the object in front of them (e.g., artwork and diorama, respectively). They were asked to pay attention to certain properties of the object, such as light, color, shape, and texture. In a fourth phase, they were instead invited to widen their gaze to encompass the whole exhibit, leaving their eyes free to move to the elements of greatest interest. Then, they were asked to close their eyes again and tune in to body sensations and breathing. Finally, the practice was concluded, with participants being invited to stretch and make any gentle movements their bodies might need. The overall duration of the Mindfulness practice was 15 min. To ensure the standardization of the protocol, a script was created and refined with the cooperation of both the instructor and the researchers. This protocol represents an adaptation of standard MBSR body scan and open monitoring practices, specifically modified for the museum environment to incorporate visual engagement with museum collections.

### 2.5. Data Analysis

All analyses were conducted using SPSS Statistics version 28.0.1.1 (IBM SPSS Statistics for Windows, Armonk, NY, USA: IBM Corp). Statistical significance was set at *p* < 0.05 (two-tailed) for all tests.

Primary outcome analysis: We used paired-samples *t*-tests to compare pre-intervention (T1) and post-intervention (T2) scores on the State Anxiety Inventory (SAI) and Visual Analog Scales (VAS) for mood, stress, mental clarity, contentment, calmness, and restlessness. Cohen’s d was used to estimate effect sizes for all comparisons, with conventional benchmarks of 0.2 (small), 0.5 (medium), and 0.8 (large) effects. Visual inspection and one-sample Kolmgorov–Sminorv test was used to control variables’ normal distribution. No significant violations were detected.

Change score computation: To quantify intervention response, we computed anxiety change scores as Delta_SAI = SAI_pre − SAI_post, where higher positive values indicate greater anxiety reduction. This scoring direction was chosen to facilitate interpretation (higher scores = better outcomes).

Individual difference analyses: To explore which participant characteristics were associated with intervention response, we conducted the following:

(a) Pearson bivariate correlations between Delta_SAI and baseline characteristics (Big Five personality traits, trait anxiety, perceived stress, interest in art, interest in science). Correlations were computed for the total sample and separately for each museum setting.
(b) Multiple linear regression models with Delta_SAI as the dependent variable. For the total sample, we entered theoretically relevant predictors (openness, art interest, science interest) identified from the correlation analyses. For each museum separately, we entered variables showing associations with Delta_SAI at *p* < 0.20 in the correlation analyses, allowing us to identify setting-specific predictors while controlling for other factors.

Separate versus combined museum analyses: We also analyzed data from the two museums (Modern Art Gallery, Natural History Museum) separately rather than in a single combined model for three reasons. First, this study was designed as a protocol feasibility study across different contexts, not as a comparative effectiveness trial. Second, the two museums were purposively selected to represent different environmental contexts (art vs. science) rather than randomized conditions. Third, separate analyses allow us to identify context-specific predictors of intervention response, providing actionable information for museums implementing the protocol in their specific setting. Because art and science museums engage different cognitive–emotional processes and serve populations with different interests and backgrounds, we expected that different individual characteristics would predict intervention response in each setting. Identifying these setting-specific patterns provides practical information for museums implementing the protocol, allowing art museums to understand which of their visitors may experience optimal benefits, and science museums to do likewise for their distinct audiences.

We conducted an independent samples *t*-test comparing Delta_SAI between museums to verify that overall intervention effects were comparable across settings. However, we did not test formal statistical interactions (Museum × Trait) as the study was not designed and powered for such regression analyses examining mediation effects.

These exploratory analyses should be interpreted as hypothesis-generating rather than definitive, given the modest sample sizes and the kind of statistical tests conducted. Future research with larger samples and pre-registered hypotheses should test whether the patterns we observe here can be replicated.

Missing data: Our procedure and the attention dedicated to data collection mostly prevented missing data. However, cases with missing data on specific measures were excluded from analyses involving those measures using listwise deletion. The final sample sizes for each analysis are reported in the Results Section.

## 3. Results

### 3.1. Descriptive Statistics

We performed descriptive statistics to provide a comprehensive picture of the personal and psychological characteristics of our participants. Table 2 reports participants’ habits concerning museum attendance, while Table 3 reports to what extent participants consider themselves art or science lovers. Most of the participants reported visiting museums regularly, and they also reported being interested in the subject of art or science.

Regarding the psychological traits, Table 4 reports participants’ trait anxiety levels and scores on the Perceived Stress Scale (PSS).

Our sample reported moderate self-evaluated levels of stress and anxiety. Since we ran our study in two different settings (GAM and NHM), we compared the respective trait variables. No significant differences were found between the two groups for any of the trait variables measured, as assessed by independent samples *t*-tests (all *p* > 0.05). This indicates that the two groups were comparable at baseline on key psychological characteristics.

### 3.2. Analyses to Assess Changes in Anxiety Levels After Attending the Mindfulness Session

We assessed outcomes immediately after the intervention, capturing acute effects. To assess whether the Mindfulness practice had an effect on state anxiety or mood levels, paired-sample *t*-tests were conducted for the measures of interest including SAI and VASs (see Table 5).

**Figure 2:**
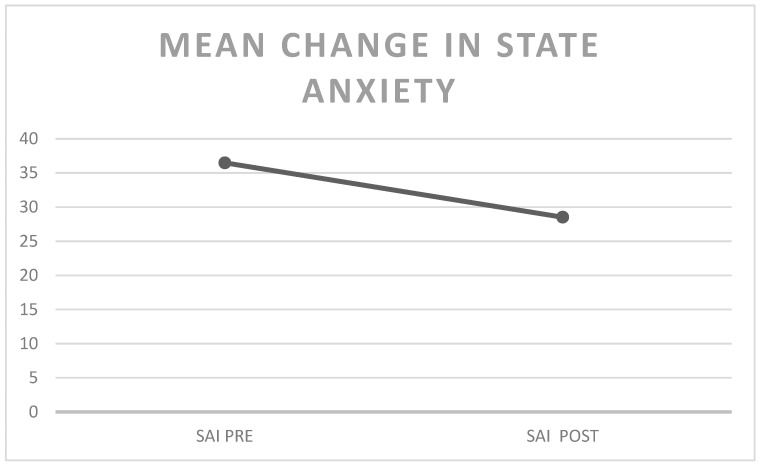
SAI scores recorded before (**left**) and after (**right**) the Mindfulness practice in the total sample.

All the variables considered showed significant changes in the expected direction (wellbeing improvement). In particular, our main variable (state anxiety) showed a mean decrease of 7.962 points on the SAI scores (see Figure 2).

For the total sample, correlation analyses were used to examine associations between anxiety reduction (Delta_SAI) and participant characteristics. Interest in art (r = 0.262, *p* < 0.05) and interest in science (r = 0.225, *p* < 0.05) showed significant positive correlations with Delta_SAI, indicating that individuals with more cultural interests experienced larger anxiety reductions. Openness to experience showed a moderate association (r = 0.211, *p* = 0.062) that approached but did not reach statistical significance.

To examine whether these associations held when controlling for other factors, we conducted a multiple linear regression with Delta_SAI as the outcome and art interest, science interest, and openness as simultaneous dependent variables.

This model explained 9.5% of variance in anxiety reduction (R^2^ = 0.095, F(3,75) = 2.63, *p* = 0.056), but no individual predictor reached statistical significance in this combined model, likely due to multicollinearity among the predictors or insufficient power to detect effects in the presence of other variables.

Given that (a) the two museums represent fundamentally different contexts, and (b) theoretical considerations suggest that different mechanisms may operate in art versus science settings, we conducted separate exploratory analyses for each museum to identify context-specific predictors.

To verify that overall intervention effects were comparable across the two museum settings, we conducted an independent samples *t*-test comparing Delta_SAI between museums. No significant difference was found (t(77) = −1.115, *p* = 0.225), indicating that both museum experiences yielded similar magnitudes of anxiety reduction overall.

However, given our goal of identifying context-specific factors that predict intervention response in different museum types, we present exploratory correlation and regression analyses separately for each setting below. This approach provides practical information for museums implementing the protocol in similar contexts, allowing them to understand which visitor characteristics may be associated with better outcomes in their specific environment.

**Figure 3:**
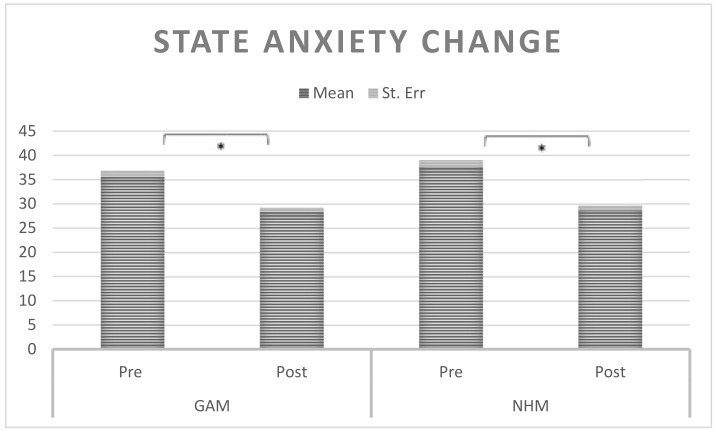
Histograms of the SAI scores recorded before and after the Mindfulness practice in the two museums. * Significant difference, *p* < 0.05.

As described in the Introduction, we analyzed each museum setting separately to identify context-specific predictors of intervention response rather than to compare overall effectiveness. The independent samples *t*-test reported above (*p* = 0.225) confirms that the two museums yielded statistically equivalent overall anxiety reductions, addressing the question of whether the protocol “works” in both contexts. As showed in Figure 3, statistical differences in state anxiety were present in both museums.

#### 3.2.1. Art Museum (MAG): Context-Specific Predictors

The *t*-tests revealed multiple significant results for SAI, mood, stress, contentment, calmness, and restlessness (see Table 6). Table 6 and Table 7 present the descriptive statistics for all outcome measures in the art museum (MAG) and the pre/post effects for all the dependent variables, respectively.

In detail, anxiety levels, stress, and restlessness were significantly decreased after the Mindfulness practice, while mood, contentment, and calmness were significantly increased (see Table 7). No significant results emerged for mental clarity levels. The effect sizes were large for anxiety (d = 1.07), mood (d = −1.1), and stress (d = 0.97), and medium for contentment (d = −0.63), calmness (d = −0.71), and restlessness (d = 0.63), indicating clinically meaningful changes.

**Figure 4:**
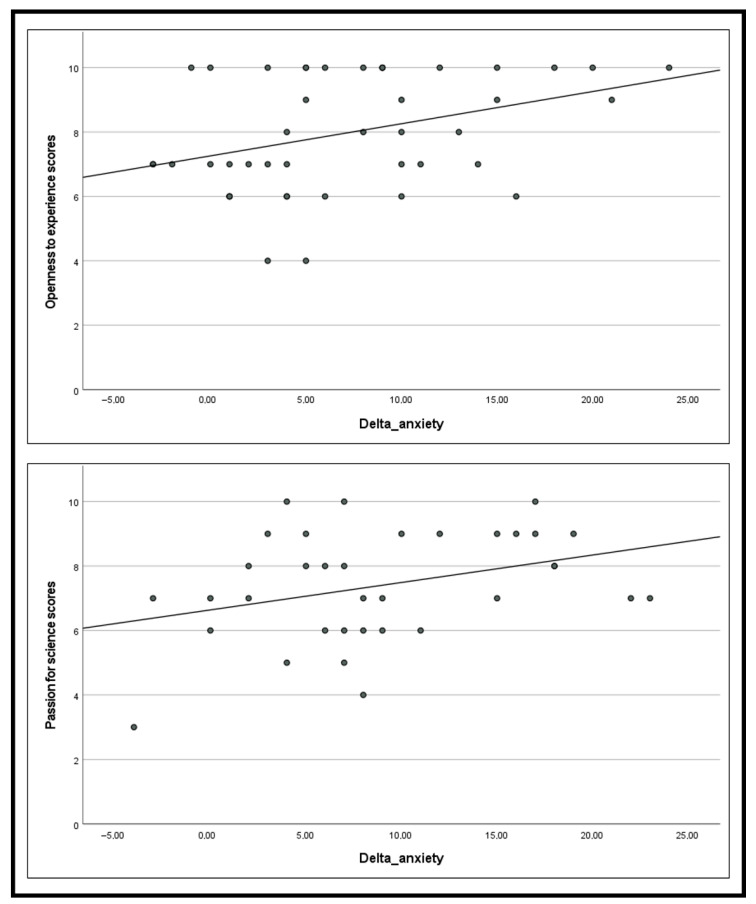
Scatterplot of Delta_anxiety vs. openness to experience scores for MAG participants (**up**) and vs. passion for science for NHM participants (**down**).

Correlation analyses revealed that greater anxiety reduction (Delta_SAI) was significantly associated with higher openness to experience (r = 0.37, *p* < 0.05). Moderate positive associations that did not reach statistical significance were observed with perceived stress (r = 0.215), interest in art (r = 0.234), and neuroticism (r = 0.213), suggesting potential trends worthy of investigation in larger samples (see Figure 4).

To examine these relationships while controlling for intercorrelations among predictors, we conducted a multiple linear regression with Delta_SAI as the dependent variable and openness, perceived stress, art interest, and neuroticism as simultaneous predictors. This model explained 20.1% of variance in anxiety reduction (R^2^ = 0.201, F(4,39) = 2.45, *p* = 0.063). Within this model, only openness to experience was a significant unique predictor (B = 1.318, SE = 0.594, β = 0.352, t = 2.221, *p* = 0.032), indicating that individuals higher in openness experienced approximately 1.3 additional points of anxiety reduction per unit increase in openness, controlling for other factors. The other variables did not contribute significant unique variance when openness was in the model.

These findings suggest that in art museum settings, personality characteristics related to esthetic receptivity and cognitive flexibility may facilitate a deeper engagement with the mindfulness practice.

#### 3.2.2. Science Museum (NHM): Context-Specific Predictors

The *t*-tests revealed significant differences for all the variables (see Table 8). Table 8 and Table 9 present the descriptive statistics for all outcome measures in the science museum (NHM) and the pre/post effects for all the dependent variables, respectively.

In detail, anxiety levels, stress, and restlessness were significantly decreased after the Mindfulness practice, while mood, mental clarity, contentment, and calmness were significantly increased (see Table 9). Notably, the effect sizes were large for all variables except mental clarity (d = −0.54, medium effect), with particularly strong effects for anxiety (d = 1.27) and stress (d = 1.44).

Thus, the Mindfulness practice was able to significantly reduce state anxiety in both museum settings (see Figure 2). No statistical differences were present between the two settings.

Correlation analyses revealed that greater anxiety reduction (Delta_SAI) was significantly associated with higher interest in science (r = 0.351, *p* < 0.05), indicating that participants with stronger science interest experienced larger decreases in anxiety.

A linear regression confirmed this association: science interest significantly predicted Delta_SAI (R^2^ = 0.199, F(1,33) = 8.18, *p* = 0.007; B = 1.400, SE = 0.489, β = 0.446, t = 2.86, *p* = 0.007). This model indicates that for each one-unit increase in science interest (on the 1–10 scale), participants experienced an average 1.4-point greater reduction in state anxiety. The model explained approximately 20% of the variance in intervention response (see Figure 4).

These findings suggest that in science museum settings, domain-specific interest and intellectual engagement with exhibit content may facilitate more effective mindfulness practice.

## 4. Discussion

This protocol development study demonstrates that a standardized MBSR-adapted intervention can be successfully implemented in museum settings with large, immediate effects on visitor anxiety and wellbeing. The 15 min sessions produced anxiety reductions (Cohen’s d = 1.07–1.27) that exceeded those typically observed in full 8-week MBSR programs (d ≈ 0.5; Khoury et al., 2015; de Vibe et al., 2017), suggesting that museums may provide particularly conducive environments for mindfulness practice.

Whereas our single-group pre–post design limits definitive causal inference, several patterns provide converging evidence that observed changes reflect genuine intervention effects rather than measurement artifacts or non-specific factors. First, the consistency of large anxiety reductions across two fundamentally different museum contexts, despite marked differences in sensory environments, exhibit content, and cognitive demands, suggests robust intervention effects transcending setting-specific characteristics. If museum atmosphere alone drove results, we would expect greater variability between such divergent environments. Second, differential effects on secondary outcomes demonstrate a specificity that is inconsistent with global response bias: mental clarity improved significantly only in the science museum (*p* = 0.003, d = −0.54) with no change in the art museum (*p* = 0.806, d = −0.04), indicating that participants did not uniformly report improvements across all measures due to social desirability. Third, context-specific predictors emerged that align with theoretical mechanisms: openness to experience predicted outcomes in the art museum (r = 0.37, *p* < 0.05), and science interest predicted outcomes in the science museum (r = 0.35, *p* < 0.05). Such theoretically coherent, setting-specific patterns would not emerge from regression to the mean or placebo effects, which operate randomly without systematic associations between baseline characteristics and treatment response.

Additionally, the magnitude and temporal characteristics of our effects argue against simple alternative explanations. A significant share of our participants were experienced museum-goers (54% visiting > 6 times annually) yet demonstrated anxiety reductions exceeding those reported in studies of museum visits alone (Binnie, 2010: d = 0.5). Natural state anxiety fluctuations over 15–30 min rarely exceed d = 0.2 with mean changes of 2–3 SAI points (Grös et al., 2007), but our observed changes (7–9 points) were three times larger. Brief mindfulness interventions in other settings show smaller effects (Howarth et al., 2019: g = 0.33; Sparacio et al., 2024: d = 0.20–0.30). This convergence of evidence (cross-context consistency, differential secondary outcomes, theory-coherent predictors, effect sizes exceeding comparison conditions) strengthens our confidence that the mindfulness practice itself, rather than non-specific factors, drove observed improvements. Nonetheless, randomized controlled trials remain essential for establishing causality and identifying active ingredients.

### 4.1. Corroboration of Study Hypotheses

Having established confidence in our findings’ validity, we now evaluate our a priori hypotheses against the observed results. Hypothesis 1 predicted significant anxiety reduction in both museum settings. This hypothesis received strong support: both the art museum (t(43) = 7.13, *p* < 0.001, d = 1.07) and science museum (t(34) = 7.46, *p* < 0.001, d = 1.27) demonstrated large, statistically significant pre–post reductions in state anxiety, with effect sizes substantially exceeding our expectations based on conventional MBSR literature. The consistency of effects across fundamentally different museum contexts provides particularly compelling support for H1, suggesting the protocol’s core mechanisms operate reliably regardless of exhibit content.

Hypothesis 2 predicted improvements across multiple wellbeing dimensions. This hypothesis received partial support with nuanced patterns. Both museums showed significant improvements in mood (art: d = −1.10, *p* < 0.001; science: d = −0.86, *p* < 0.001), perceived stress (art: d = 0.97, *p* < 0.001; science: d = 1.44, *p* < 0.001), contentment (art: d = −0.63, *p* < 0.001; science: d = −0.80, *p* < 0.001), calmness (art: d = −0.71, *p* < 0.001; science: d = −1.03, *p* < 0.001), and restlessness (art: d = 0.63, *p* < 0.001; science: d = 0.91, *p* < 0.001). However, mental clarity showed differential patterns discussed under H4 below. The breadth of significant improvements across affective, somatic, and cognitive domains demonstrates that the intervention’s effects extended well beyond anxiety reduction alone, supporting a holistic wellbeing impact.

Hypothesis 3 predicted context-specific individual difference predictors. This hypothesis received strong support with remarkable specificity. In the art museum, openness to experience significantly predicted anxiety reduction (r = 0.37, *p* < 0.05) and remained the only significant predictor in multiple regression (β = 1.32, *p* = 0.032), whereas science interest showed no association. Conversely, in the science museum, science interest significantly predicted anxiety reduction (r = 0.35, *p* < 0.05; β = 1.40, *p* = 0.007), whereas openness showed no significant relationship. This double dissociation, where each predictor operated exclusively in its theoretically predicted context, provides particularly strong evidence for H3. The pattern argues against alternative explanations such as general response bias or non-specific personality effects, instead supporting the hypothesis that different psychological processes mediate intervention response in different museum environments.

Hypothesis 4 predicted differential effects across museum types, specifically stronger affective engagement in art museums (H4a) and greater mental clarity improvements in science museums (H4b). H4a received mixed support: while both museums produced strong affective improvements (mood, contentment, calmness), the art museum did not demonstrate significantly larger effects than the science museum on these dimensions, contrary to predictions. H4b, however, received clear support: mental clarity improved significantly in the science museum (t(34) = 3.17, *p* = 0.003, d = −0.54) but showed no change in the art museum (t(43) = 0.25, *p* = 0.806, d = −0.04). This differential effect on mental clarity, the only outcome variable showing such museum-specific patterns, provides compelling evidence that different museum contexts activate distinct cognitive-affective pathways. The science museum’s emphasis on systematic observation and knowledge structures appears to promote focused, analytical mind states, while the art museum’s emphasis on emotional resonance and esthetic experience may activate different psychological processes not captured by our “mental clarity” measure.

In summary, three of four primary hypotheses (H1, H3, H4b) received strong empirical support, H2 received partial support, and H4a received mixed support. Most importantly, the pattern of findings demonstrates theoretical coherence: the intervention works across contexts (H1), produces broad wellbeing benefits (H2), but operates through context-specific mechanisms involving different individual characteristics (H3) and cognitive-affective pathways (H4b). This combination of consistent primary outcomes with differential mediating processes hints at both the protocol’s robustness and its theoretical sophistication.

These findings also contribute to the growing evidence base supporting “social prescribing” initiatives, where healthcare providers recommend cultural activities, and museum visits in particular, as part of treatment plans (Thomson et al., 2018).

The standardized protocol we developed provides a concrete framework that museums can implement on reasonable evidence-based grounds. This is particularly relevant given the increased focus on mental health following the COVID-19 pandemic and the recognized need for accessible, community-based interventions (Fischer-Grote et al., 2024; American Alliance of Museums, 2024).

### 4.2. Context-Specific Predictors of Intervention Response

We now examine in more detail the specific mechanisms and contextual factors that predicted outcomes across different museum settings. Our findings demonstrate that both art and science museums can effectively serve as venues for anxiety reduction, consistent with previous research (Mastandrea et al., 2019; Šveb Dragija & Jelinčić, 2022; French et al., 2020), revealing distinct psychological pathways operating in each context.

In the art museum, anxiety reduction was significantly predicted by openness to experience (r = 0.37, *p* < 0.05), which remained the only significant predictor in multiple regression (β = 1.32, *p* = 0.032) after controlling for perceived stress, art interest, and neuroticism. This personality trait, characterized by creativity, curiosity, and willingness to explore new experiences (Baehr, 2011), signals an individual’s propensity toward esthetic engagement and cognitive flexibility. The correlation between openness and intervention response suggests that individuals with greater aesthetic receptivity can more readily engage with the rich visual complexity of artworks during mindful observation: processing colors, compositional elements, and emotional content in ways that deepen the mindfulness practice. This finding aligns with research demonstrating that open-minded individuals show enhanced absorption capacity and are more likely to experience transformative aesthetic encounters (Kuijpers et al., 2019).

These results partially parallel those of de Vibe et al. (2015), who found that personality traits moderated MBSR effectiveness in medical students, though they identified neuroticism and conscientiousness as primary moderators rather than openness. The divergence may reflect different intervention contexts: clinical MBSR programs for stressed students versus brief museum-based sessions for community participants. Our findings suggest that in esthetically rich environments, openness specifically facilitates the emotion-centered pathway through which mindfulness practice reduces anxiety, a mechanism distinct from the stress-buffering effects neuroticism moderation might provide in clinical contexts.

In contrast, the science museum showed a different pattern: anxiety reduction was significantly predicted by interest in science (r = 0.35, *p* < 0.05; β = 1.40, *p* = 0.007), explaining approximately 20% of variance in intervention response. Here, domain-specific engagement and intellectual curiosity, rather than broad personality traits, predicted outcomes. Participants with greater science knowledge and interest could engage more meaningfully with the dioramas’ biological and ecological content, using their intellectual scaffolding to sustain focused attention during mindfulness practice. This cognitive-analytical pathway differs qualitatively from the emotion-centered pathway observed in the art museum.

The differential effects on mental clarity further support distinct mechanisms across settings. Mental clarity improved significantly only in the science museum (*p* = 0.003, d = −0.54), with no change in the art museum (*p* = 0.806, d = −0.04). We suggest that scientific exhibits promoted focused, analytical attention states, while figurative art elicited more emotionally engaged states. This pattern demonstrates that participants were not simply reporting uniform improvements due to demand characteristics but rather experiencing genuinely different psychological processes depending on museum context.

These findings reveal that intervention effectiveness depends not solely on individual characteristics or environmental features alone, but on the interaction between visitor attributes and environmental affordances. Art museums may optimally serve visitors seeking emotional regulation through esthetic engagement, activating pathways requiring cognitive flexibility and openness. Science museums may better serve those preferring structured, knowledge-based exploration, activating pathways requiring domain interest and analytical focus. This insight has important implications for personalizing museum-based mental health programming.

However, our exploratory analyses should be interpreted as hypothesis-generating rather than definitive, given the modest sample sizes (*n* = 35–44 per museum) and possible risk of Type I error. The differential predictors we identified (openness in art museums, science interest in science museums) represent promising patterns requiring confirmation through independent replication with larger samples, pre-registered hypotheses, and appropriate statistical safeguards before informing practice or policy.

### 4.3. Implications for Theory

Our findings contribute to the theoretical understanding of mindfulness mechanisms and environmental moderators of intervention effectiveness. The remarkable effect sizes observed (d = 1.07–1.27) in single 15 min sessions raise important questions about dose–response relationships in mindfulness interventions. Standard MBSR programs involve 20–30 h of practice over 8 weeks (Kabat-Zinn, 1990) yet produce smaller average effects (d ≈ 0.5) in non-clinical populations. Controlled trials are needed for direct comparison, but our preliminary findings already suggest that environmental context may amplify intervention effects beyond what practice duration alone predicts.

Several theoretical mechanisms may explain this enhancement. First, museums provide naturalistic attentional anchors: artworks and specimens offer visually complex, esthetically engaging stimuli that facilitate sustained attention without requiring effortful concentration. Unlike breath-focused meditation, which novices often find difficult, mindful observation of compelling cultural objects may reduce mind-wandering through inherent stimulus interest. Second, museums activate curiosity and openness mindsets that may synergize with mindfulness practice. The act of entering a museum signals permission to explore, wonder, and engage aesthetically: all states that are consonant with mindfulness’s non-judgmental, present-moment awareness. Third, museums provide implicit social permission for contemplative experience. Unlike clinical settings where treatment-seeking may activate anxiety, or workplace settings where productivity norms dominate, museums culturally sanction the freedom of slowing down and observing carefully.

These environmental affordances align with theories of embodied and situated cognition, which emphasize how physical and social contexts shape psychological processes (Varela et al., 2017). Mindfulness may not function identically across all environments; rather, specific contexts may activate distinct pathways or amplify core mechanisms. Our finding of differential predictors across museum types supports this view: the same standardized protocol engaged different psychological processes (emotion-centered vs. cognitive-analytical) depending on exhibit content and individual characteristics.

Our results also offer insights into cultural participation and wellbeing. Previous research has documented associations between museum visits and health outcomes (Cuypers et al., 2012; Fancourt & Steptoe, 2019) without clarifying the underlying mechanisms. Our findings suggest that structured contemplative practices may transform passive museum exposure into active wellbeing interventions. The integration of mindfulness with cultural engagement creates synergistic benefits: mindfulness enhances esthetic appreciation and learning, and cultural content provides meaningful attentional objects that sustain mindfulness practice. This bidirectional relationship positions museums as unique venues where mental health promotion and cultural enrichment occur simultaneously, advancing neither at the expense of the other.

Finally, our identification of context-specific predictors contributes to personalized intervention theory. Instead of seeking universally effective approaches, our findings suggest that museums should focus upon visitor-environment fit: matching individual characteristics (personality, interests) to appropriate museum contexts (art, science) and programming types. This aligns with precision medicine frameworks that emphasize tailored interventions over one-size-fits-all approaches.

### 4.4. Implications for Practice and Policy

These findings offer concrete guidance for museums, healthcare systems, and policymakers interested in museum-based mental health programming. This protocol development study represents Phase 1 of the ASBA project, testing the mindfulness component specifically. Future ASBA phases will systematically evaluate other evidence-based approaches (Art Therapy for processing difficult emotions through creative expression; Visual Thinking Strategies for developing observational and interpretive skills; Nature + Art for combining biophilic and esthetic engagement) to build a portfolio of museum-based interventions addressing diverse visitor needs, preferences, and therapeutic goals. This multi-method approach recognizes that different individuals may respond optimally to different modalities.

For museums implementing wellbeing programs, the standardized ASBA mindfulness protocol provides a replicable framework requiring minimal resources. Sessions need only 15 min, use existing gallery spaces during regular operating hours, and do not require specialized equipment beyond chairs. The primary resource is access to a certified MBSR instructor, which many communities already have through hospital wellness programs, universities, or private practitioners. Museums can implement this protocol immediately while awaiting definitive efficacy data from randomized trials, as the intervention poses minimal risk and preliminary evidence suggests substantial benefits.

Moreover, the context-specific predictors may inform programming design. Art museums may particularly benefit visitors high in openness to experience, suggesting marketing to creative, curious populations or designing multiple tracks (e.g., guided mindfulness for less open visitors, self-directed practice for highly open visitors). Science museums should consider the science interest of visitors when designing sessions, potentially offering thematic pathways matching different interest areas (biology, geology, astronomy) to optimize domain engagement. Both museum types might benefit from brief pre-session assessments helping visitors select optimal programs based on personality and interests.

Regarding social prescribing initiatives, our findings contribute to the evidence base for healthcare–museum partnerships. Several countries have pioneered museum prescription programs, but these initiatives have lacked standardized protocols and systematic outcome evaluation. The ASBA protocol addresses this gap, providing physicians with a specific, evidence-informed intervention to recommend in place of generic museum visits. Healthcare systems evaluating museum programming as adjunct or alternative treatment now have preliminary data suggesting that brief, single-session interventions may produce effects comparable to multi-session clinical programs, with implications for cost-effectiveness and accessibility.

The clinical significance of our effect sizes merits some attention. The observed anxiety reductions represent participants moving from moderate anxiety levels (mean SAI scores 35–38) to substantially lower, near-normal ranges (mean SAI 28–29) within a single session. This magnitude crosses established thresholds for meaningful symptom improvement and approaches anxiety levels typical of non-clinical populations. Single-session improvements proved comparable to multi-week cognitive-behavioral therapy protocols for anxiety disorders, highlighting the remarkable therapeutic potential of combining mindfulness practice with culturally rich environments.

For public health policy, museums represent widespread, trusted cultural infrastructure already serving diverse populations. Unlike specialized mental health services requiring referrals and potentially carrying stigma, museums qualify as culturally normalized venues for wellbeing activities. The ASBA framework provides a model for transforming existing community assets into mental health resources without requiring new infrastructure. This approach directly addresses persistent post-pandemic mental health needs (Fischer-Grote et al., 2024; American Alliance of Museums, 2024) through accessible, community-integrated programming.

Museums that implement ASBA programming simultaneously fulfill mental health promotion and cultural education missions. Differently from clinical interventions imported wholesale, our protocol integrates mindfulness with curator-led discussion, ensuring that cultural content remains central. This integration likely contributed to institutional buy-in and may amplify therapeutic effects through meaning-making and intellectual engagement, that is, benefits highly characteristic of museum settings.

Regarding scalability, our protocol’s standardization enables systematic implementation and evaluation across diverse museum types globally. Museums worldwide possess the necessary infrastructure (exhibition spaces, educated staff, community trust) to deliver this intervention. Training requirements involve primarily instructor certification (widely available through MBSR professional training programs) and brief curator orientation to the inquiry phase. The protocol’s documented effectiveness across both art and science museums suggests broad applicability, though replication in other museum types (history, children’s, contemporary art, etc.) would further establish its generalizability.

### 4.5. Strengths and Limitations

This study’s strengths include the development of a fully standardized, replicable protocol with detailed documentation enabling widespread implementation; cross-context validation that demonstrates consistent effects across art and science museums; use of validated outcome measures with large, clinically meaningful effect sizes exceeding published benchmarks; adequate statistical power (>0.99) for primary outcomes; identification of theoretically coherent, context-specific predictors; and ecological validity through the implementation in operating museums during regular hours with community-recruited participants.

However, several limitations call for careful and cautious interpretation. Most significantly, our single-group pre–post design prevents definitive causal inference. Without randomized control groups, we cannot conclusively determine whether observed changes resulted from the mindfulness practice itself, museum environmental factors, social aspects of group participation, or their interactions. The convergence of internal evidence (cross-context consistency, differential secondary outcomes, theory-coherent predictors, effect sizes exceeding comparison conditions) strengthens confidence in intervention-specific effects, but controlled trials remain nevertheless essential.

Measurement limitations include exclusive reliance on self-report instruments that are potentially vulnerable to social desirability bias, demand characteristics, and response shift. Although differential effects across outcomes argue against uniform positive responses, future research should incorporate objective measures (e.g., salivary cortisol, heart rate variability, blood pressure) and behavioral observations to triangulate findings. We assessed only immediate pre–post changes, providing no information about effect duration or whether brief interventions prompt lasting behavior changes (continued museum visitation, adoption of mindfulness practice, etc.). The observed anxiety reductions represent acute responses occurring within the intervention context. Whether these benefits persist beyond the museum visit, extend into daily life, or accumulate with repeated sessions remains unknown. Follow-up studies tracking participants over weeks to months are needed for this purpose.

The study’s sample limitations include its modest size (*n* = 79), which is adequate for preliminary research but insufficient for complex subgroup or mediation analyses. We did not conduct a priori power analysis, which represents a planning limitation, though the large observed effect sizes and narrow confidence intervals suggest our sample provided adequate power for detecting the primary intervention effects. We also have substantial gender imbalance with 86% female participants. This gender distribution reflects common patterns in mindfulness and wellness program recruitment (Khoury et al., 2015) but limits conclusions about intervention effectiveness in men. Although our analyses found no gender differences in treatment response, the small number of male participants (*n* = 11 total) provides insufficient statistical power to detect meaningful gender effects. In the light of documented gender differences in anxiety prevalence, symptom expression, help-seeking behavior, and treatment response (McLean et al., 2011), future studies should employ targeted recruitment strategies to achieve better gender balance. Understanding whether the intervention works equally well for men and women has important implications for program marketing, implementation, and public health impact. The predominance of women may also reflect broader patterns of museum visitation and cultural engagement, suggesting that additional outreach may be needed to engage male participants in museum-based wellness programming.

Our convenience sample recruited through university, government, and museum websites likely attracted culturally engaged individuals (54% visited museums > 6 times annually), potentially limiting generalizability to less culturally active populations. We excluded individuals with current psychiatric diagnoses, precluding conclusions about clinical populations, though moderate baseline anxiety levels (SAI 35–38) suggest promising effectiveness for subclinical symptomatology.

Environmental and procedural variability arose from conducting research in operating museums, introducing ambient noise, varying lighting conditions, and uncontrollable group dynamics. This trade-off prioritized external validity and real-world applicability over experimental control. Exhibit content differed substantially between museums (romantic paintings vs. natural history dioramas), but the consistency of large effects despite contextual variations suggests that the protocol is robust. Statistical limitations include our exploratory predictor analyses involving multiple tests without pre-registration, carrying the risk of Type I errors. Although theory-coherent observed patterns suggest that effects are genuine rather than statistical noise, it is essential to replicate them with pre-registered hypotheses.

The validity of the results remains limited to the Italian urban context of the study. Museum cultures, visitor expectations, and mindfulness receptivity vary substantially across countries and regions. The protocol must be further tested across diverse cultural contexts, geographic regions, museum types (history, contemporary art, children’s museums, etc.), and implementation models (individual vs. group formats, varying session durations, different instructor types) before claiming its universal applicability.

These limitations place boundaries on our current conclusions and set a promising agenda for subsequent research. This study was not designed to provide definitive proof of efficacy: that remains the goal of future randomized controlled trials informed by our work. We aimed to build a foundation, which requires different methodological priorities than hypothesis testing. Protocol development necessarily occurs in naturalistic settings with real participants facing real constraints. Perfect control would compromise external validity and provide false confidence about transportability to practice settings.

The appropriate response to these limitations is neither dismissal of our findings nor uncritical acceptance, but rather proportional confidence calibrated to the evidence. We have strong preliminary support for museum-based Mindfulness, sufficient to justify investment in rigorous trials but insufficient to claim established efficacy. This represents exactly where Stage 1b intervention development research should position the field: at the threshold of definitive evaluation, with a clear path forward.

### 4.6. Future Research Directions

Future randomized controlled trials should compare museum-based mindfulness against multiple control conditions to isolate active ingredients: museum visit alone (to determine whether structured mindfulness adds value beyond museum exposure); mindfulness in neutral settings (to assess whether museums provide uniquely favorable contexts); curator-led discussion without mindfulness (to separate mindfulness-specific effects from social learning and esthetic engagement); and waitlist control (to establish overall efficacy). Factorial designs examining interactions between setting type, individual characteristics, and group composition would further clarify which implementation strategies are most appropriate.

Essential methodological enhancements include longer follow-up periods (1-month, 3-month, 6-month) assessing sustainability and behavior change; objective outcome measures such as biomarkers and behavioral observations; larger, more diverse samples enabling subgroup analyses by gender, age, baseline severity, and cultural background; inclusion of participants with diagnosed anxiety disorders (with appropriate clinical oversight) to test clinical effectiveness; and dose–response investigations comparing single-session to multi-session protocols.

Implementation science research should examine barriers and facilitators to broader dissemination, cost-effectiveness compared to traditional mental health services, training requirements for museum staff delivering the protocol, and adaptations needed for specific museum types and populations. Cross-cultural replication in North American, Asian, African, and other European contexts would provide precious cross-cultural insight. Likewise, testing in diverse museum types such as history, contemporary art, science centers, and children’s museums would probe the protocol’s flexibility.

Mechanistic research should dig deeper into the hypothesized pathways: Does mindful attention to museum artifacts increase esthetic absorption? Do museums activate curiosity mindsets synergizing with mindfulness? What specific exhibit characteristics (complexity, familiarity, emotional valence) optimize mindfulness practice? Comparative studies examining museum-based mindfulness against standard clinical MBSR, other museum-based interventions (art therapy, visual thinking strategies), and non-museum community-based mindfulness would be very helpful to contextualize our findings within the broader intervention landscape and test whether the apparently larger acute effects we observed replicate under controlled conditions.

Finally, personalization research should systematically test whether matching visitors to contexts based on personality and interests improves outcomes beyond main effects, using machine learning approaches, possibly also collecting biological data such as electro-cortical activity (Vanutelli et al., 2025), to identify complex patterns predicting optimal visitor-environment fit. Such research would advance precision approaches to museum-based mental health programming, maximizing public health impact and at the same time respecting individual differences.

## 5. Conclusions

As museums worldwide increasingly embrace roles as community health resources, standardized protocols enable scientifically grounded practice. The ASBA mindfulness protocol tested here represents the first component of a broader initiative to establish museums as credible partners in the creative health ecosystem. We developed and tested a standardized 15 min MBSR-adapted mindfulness protocol across art and science museum settings, demonstrating feasibility and preliminary efficacy for anxiety reduction in healthy community adults. The protocol integrates contemplative practice with cultural engagement through curator collaboration, offering museums a replicable framework requiring minimal resources beyond instructor training.

Key findings establish that (1) both museum types produced large, immediate anxiety reductions (d = 1.07–1.27) with broad wellbeing improvements; (2) intervention response was predicted by distinct factors in each setting (openness to experience in art museums, science interest in science museums), suggesting the existence of context-specific mechanisms on top of generic museum effects; and (3) differential cognitive outcomes (mental clarity improvement only in science museums) indicate that qualitatively different psychological processes are at work across environments.

These results position the ASBA mindfulness protocol as ready for randomized controlled trials to establish causality, assess sustainability, and optimize targeting strategies. Museums worldwide can immediately implement this evidence-informed approach and contribute data toward a more rigorous, comprehensive evaluation. As cultural institutions increasingly serve public health functions, standardized protocols enable scientifically grounded practice, turning wellbeing programming from aspirational to evidence-based. Future research should examine comparative effectiveness, long-term outcomes, and personalization algorithms matching visitor characteristics to optimal museum contexts and intervention types to fully establish museums as a key resource for public health.

## Figures and Tables

**Table 1:** Sample characteristics by museum setting.

Museum	N	Gender	Gender (%)	Age Range	Mean Age	SD
Modern Art Gallery (MAG)	44	Female	84.1	18–71	45.16	16.59
		Male	15.9			
Natural History Museum (NHM)	35	Female	88.6	21–73	48.17	16.93
		Male	11.4			
Total	79	Female	86.1	18–73	46.49	16.70
		Male	13.9			

**Table 2:** Frequencies of average annual museum visits for the two samples.

Annual Visits	Frequency	%	Cumulative %
>6	43	54.5	54.5
1–2	15	18.9	77.5
3–5	17	21.5	94.9.0
Never	4	5.1	100.0
Tot	44	100.0	

**Table 3:** Mean values of topic interest as assessed in the screening questionnaire (How interested do you think you are in art/science museums on a scale of 1 to 10? See Section 2.3.1).

Interest in	N	Min	Max	Mean	SD
Art	79	2	10	7.81	2.02
Science	79	2	10	7.14	1.81

**Table 4:** Mean values of Trait Anxiety scores (TAI), Perceived Stress scores (PSS), and self-reported stress in the last month for the two samples.

Trait Measures	N	Min	Max	Mean	SD
Trait Anxiety (TAI)	79	25	69	47.00	9.102
Perceived Stress (PSS)	79	7	37	19.61	6.217
Stress_last month (0–10)	79	2	10	6.97	1.811

**Table 5:** Descriptive statists and *t*-tests for paired samples of trait (pre–post) measures.

	Paired Differences				Significance
State Measures	Mean	Std. Deviation	*t*-Test	Df	*p*
SAI	7.962	6.747	10.489	78	<0.001
Mood	−1.182	1.243	−8.342	76	<0.001
Stress	2.101	1.795	10.407	78	<0.001
Mental Clarity	−0.481	1.608	−2.659	78	0.005
Contentment	−1.152	1.618	−6.328	78	<0.001
Calmness	−1.570	1.824	−7.651	78	<0.001
Restlessness	1.304	1.712	6.768	78	<0.001

**Table 6:** *t*-tests for paired samples output (MAG group) for all the dependent variables.

State Measures	Paired Differences Mean	St. Deviation	*t*	df	*p*
SAI	7.18	6.6	7.22	43	<0.001
Mood	−1.05	0.94	−7.26	41	<0.001
Stress	1.93	1.96	6.55	43	<0.001
Mental Clarity	−0.05	1.22	−0.25	43	0.403
Contentment	−0.86	1.36	−4.22	43	<0.001
Calmness	−1.21	1.67	−4.8	43	<0.001
Restlessness	1.14	1.77	4.25	43	<0.001

**Table 7:** Descriptive statistics for the variables with significant pre/post effects (MAG group) for all the dependent variables.

State Measures	Phase	N	Mean	SD	Mean SE
SAI	Pre	44	35.57	8.4	1.27
	Post	44	28.39	5.17	0.78
Stress	Pre	44	4.20	2.52	0.38
	Post	44	2.27	1.48	0.22
Restlessness	Pre	44	3.36	2.24	0.34
	Post	44	2.23	1.6	0.24
Mood	Pre	42	7.33	1.24	0.19
	Post	42	8.38	0.96	0.15
Contentment	Pre	44	7.48	1.58	0.24
	Post	44	8.34	1.16	0.18
Calmness	Pre	44	7.20	1.88	0.28
	Post	44	8.41	1.28	0.19

**Table 8:** *t*-tests for paired samples output (NHM group).

State Measures	Paired Differences Mean	St. Deviation	*t*	df	*p*
SAI	8.94	6.9	7.67	34	<0.001
Mood	−1.34	1.53	−5.18	34	<0.001
Stress	2.31	1.57	8.73	34	<0.001
Mental Clarity	−1.03	1.87	−3.25	34	0.001
Contentment	−1.51	1.85	−4.84	34	<0.001
Calmness	−2.03	1.93	−6.21	34	<0.001
Restlessness	1.51	1.63	5.48	34	<0.001

**Table 9:** Descriptive statistics for the variables with significant pre/post effects (NHM group).

State Measures	Phase	N	Mean	SD	Mean SE
SAI	Pre	35	37.66	8.35	1.41
	Post	35	28.71	5.59	0.94
Stress	Pre	35	4.51	2.16	0.37
	Post	35	2.20	1.53	0.26
Restlessness	Pre	35	3.31	2.35	0.4
	Post	35	1.80	1.37	0.23
Mood	Pre	35	7.06	1.64	0.28
	Post	35	8.40	1.01	0.17
Mental clarity	Pre	35	6.97	1.89	0.32
	Post	35	8	1.21	0.21
Contentment	Pre	35	6.49	2.11	0.36
	Post	35	8	1.28	0.22
Calmness	Pre	35	6.34	2.01	0.34
	Post	35	8.37	1.11	0.19

## Data Availability

The data presented in this study are available upon request from the corresponding author. The data are not publicly available due to privacy concerns.
